# Genetic Markers Used for Risk Stratification in Multiple Myeloma

**DOI:** 10.4061/2011/798089

**Published:** 2011-09-13

**Authors:** Priscilla Segges, Esteban Braggio

**Affiliations:** ^1^Laboratory of Molecular Biology, Bone Marrow Transplantation Center, National Cancer Institute, Rio de Janeiro, RJ 20230-130, Brazil; ^2^Comprehensive Cancer Center, Mayo Clinic Arizona, Scottsdale, AZ 85259-5494, USA

## Abstract

While no specific genetic markers are required in the diagnosis of multiple myeloma (MM), multiple genetic abnormalities and gene signatures are used in disease prognostication and risk stratification. This is particularly important for the adequate identification of the high-risk MM group, which does not benefit from any of the current therapies, and novel approaches need to be proposed. Fluorescence in situ hybridization (FISH) has been employed for establishing risk-based stratification and still remains the most used genetic technique in the clinical routine. The incorporation of gene expression profiling (GEP) in the study of MM has shown to be a very powerful test in the patient stratification, but its incorporation in clinical routine depends on some technical and logistic resolutions. Thus, FISH still remains the gold standard test for detecting genomic abnormalities and outcome discrimination in MM.

## 1. Background

Multiple Myeloma (MM) is a malignancy characterized by accumulation of clonal antibody-secreting plasma cells [1]. While no specific genetic markers are used for MM diagnosis, multiple genetic abnormalities have been associated with malignant transformation and disease progression [2–5]. The identification of genetics aberrations was greatly improved after the implementation of analytic tools capable to overcome the technical limitations related to low proliferation of the myeloma cell. Thus, several classifications have been proposed based on the identification of the genomic changes that help to discriminate between different genetic groups of MM patients [3, 6–9]. 

Overall, MM is divided into two main genetic groups: (1) the hyperdidploid group (H-MM), which can be defined mainly by the gain of odd chromosomes 3, 5, 7, 9, 11, 15, 19, and 21 and (2) the nonhyperdiploid group (NH-MM), characterized by the presence of chromosomal translocations involving the immunoglobulin H *(IgH)* locus with several chromosomal partners (4, 8, 11, 16) [10–12]. Each category includes approximately half of cases, with a very low number of overlapping cases. 

Of interest, the dissection of the genetic landscape has provided important genetic markers with demonstrated clinical and disease stratification value [5, 13–15].

## 2. Cytogenetic Prognostic Markers—FISH

### 2.1. t(4;14)(p16;q32)

This translocation affects the telomeric portion of chromosome 4p leading to the dysregulation of two protooncogenes, *FGFR3* in derivate chromosome 14 (der14) and multiple myeloma SET domain (*MMSET*) in derivative chromosome 4 (der4) [16]. The t(4;14) is seen in 15–20% of primary MM [17]. The translocation is cryptic and detectable only by FISH or reverse transcriptase—PCR [17]. 

Several groups have associated the t(4;14) with inferior outcome and more aggressive disease irrespective of the treatment modality [2, 5, 18, 19] (Table 1). It has been suggested that this group of patients can benefit from bortezomib-based therapy [20]. However, two recent studies showed that, although bortezomib-based therapy shows better results than previous therapies (vincristine, adriamycin, and dexamethasone) in patients with t(4;14), this translocation still has prognostic implications in a great group of patients treated with this drug [18, 19].

### 2.2. t(14;16)(q32;q23) and Other MAF Translocations

The t(14;16) is found in 5–7% of all MM cases [4, 5, 21]. The presence of t(14;16) has been associated with more aggressive disease and shorter survival among the patients treated with either conventional or high-dose chemotherapy [5, 6] (Table 1). The prognosis of this translocation was recently challenged by a study that suggests a neutral effect in a large series of patients [21]. Given the very low prevalence of the MAF abnormalities, the test to detect the presence of these translocations has not been universally incorporated in the clinical routine.

The upregulation of *CCND3* (cyclin D3), as a result of t(6;14)(p21;q32), is identified in only 3% of MM [5] (Table 1). Until now, there is no known clinical or prognostic information for this translocation.

### 2.3. t(11;14)(q13;q32)

This translocation results in the juxtaposition of *CCND1* proto-oncogene with the *IgH* locus and as consequence an ectopic expression of cyclin D1 [22]. Of all MM, the* t(11;14)* has been described in 15% of cases and is associated with CD20 expression, lymphoplasmacytic morphology, hyposecretory disease, and Ig light chain usage [22, 23]. 

Most studies have suggested that the presence alone of t(11;14) may confer a favorable outcome (Table 1), but this effect is not strong enough to be statistically significant (probably because of small magnitude of this translocation) [22–24]. Moreover, due to heterogeneity within patients with t(11;14) there exists a difficulty in establishing a favorable outcome for patients with this genetic aberration. For instance, the presence of K-RAS mutations in patients with t(11;14) is also more prevalent (50%) than in patients with other primary IgH translocations (10%) [25]. In addition, the presence of t(11;14) is associated with an aggressive phenotype such as plasma cell leukemia [23]. A recent study with a larger series of patient with t(4;14) has suggested that the effect of t(11;14) on prognosis remains neutral [24] (Table 1).

### 2.4. Ploidy Status

In MM, aneuploid is frequently observed [11, 12] and delineates the disease into two main genetic subtypes, H-MM and NH-MM. H-MM is more common among males, has a higher incidence of MM bone disease, and carries a more favorable outcome [6] (Table 1). Among patients with H-MM, 13 deletion and chromosome 1 abnormalities have not apparent prognostic significance but the presence of deletions of 17p13 in remains an important prognostic factor. In addition, a study showed that most of the prognostic value of H-MM was related to the gain of chromosome 5 [24, 26].

### 2.5. Deletion of 17p

The deletion of 17p13 remains the most important molecular prognostic factor in MM [5, 6, 20]. The deletion 17p13 is generally monoallelic and includes *TP53*. The abnormality is detected in only 10% of new diagnosis MM cases, but its prevalence increases in later stages of the disease. Patients with 17p13 deletions often have more aggressive and extramedullary disease (such as plasmacytomas), center nervous system involvement, and hypercalcemia [6, 27]. This abnormality is associated with a shorter survival irrespective of the treatment modality, including the novel bortezomib and IMiDs-based therapies [5, 6, 14, 27] (Table 1).

### 2.6. Chromosomes 1 and 13

Chromosome 1 abnormalities are found in almost half of MM cases [28]. There is an enrichment of genes associated with proliferation in the affected region [8]. Although the poor prognosis value of this abnormality has been recently demonstrated, its incorporation into standard clinical practice has not been implemented yet [28] (Table 1). 

The deletion of chromosome 13 is found in 50% of MM cases [4–6, 8, 29]. Although this abnormality was originally identified as negative prognostic factor in MM, several studies had proved the association of chromosome 13 monosomy with the t(4;14) [6, 30, 31]. Even in the absence of this association or other high-risk markers, the chromosome 13 alone is not a predictive of poor prognosis when identified by FISH (Table 1). On the other hand, its identification by conventional cytogenetics is a surrogate of high proliferation and is used as a poor prognostic marker [32] (Table 1).

## 3. Comprehensive Genomic Tools in MM Risk Stratification

The advent of high-resolution genomics tools provided a remarkable revolution in the analysis of MM and the identification of genomic signatures able to identify high-risk patients and to predict patient outcome [6, 8, 33]. Several high-resolution available tests provide a comprehensive analysis at the DNA (aCGH, single-nucleotide polymorphism (SNP) arrays, and whole-genome sequencing (WGS)) and RNA levels (gene expression profiling (GEP)). 

Among these technologies, the use of GEP is the most promising risk stratification tool in MM. The use of GEP has been successfully implemented in MM, and several genetic signatures have been proposed [8, 33, 34]. The most used signatures are based on the analysis of proliferation markers or in centrosome index and successfully detected the 15–20% of worse prognosis patients [35]. 

The prognostic classification, using genetic analysis as outcome discrimination, has been used in several cohorts of MM treated with the conventional and high-dose chemotherapy followed by stem cell transplant (SCT) [5, 8, 35]. Moreover, the ongoing studies involving patients with MM are focused on the use of these genetic markers provided by genetic changes, as predictors of outcome in those treated with proteasome inhibitors. Although GEP is still mainly used for research purposes, some groups have successfully implemented its use in the routine clinical care [35]. Other approaches such as aCGH and WGS have not been implemented in the clinical routine yet, being used in the research laboratory.

## 4. Conclusion

Genetic studies have played a crucial role in the determination of the risk-based stratification of MM. Nowadays, FISH and GEP are the most powerful tools for successfully identifying disease subgroups with different outcomes.

## Figures and Tables

**Table 1:** Abnormalities associated with outcome in MM and techniques used for detection.

Abnormalities	Outcome	Test
t(4;14)(p16;q32)	Poor	FISH*
t(14;16)(q32;q23)	Poor	FISH
t(6;14)(p21;q32)	Good?	FISH
t(11;14)(q13;q32)	Good/neutral	FISH
Deletion 17p13	Poor	FISH
Deletion 13	Poor	Convenlional cytogenetics
Chromosome 1 abnormalities	Poor	FISH
Hyperdiploidy	Good	FISH or FCM**
(if not associated with deletion 17p13)		

*Fluorescence in situ hybridization (FISH). **Flow cytometry.

## References

[1] Kyle RA, Rajkumar SV (2004). Drug therapy: multiple myeloma. *The New England Journal of Medicine*.

[2] Fonseca R, Barlogie B, Bataille R (2004). Genetics and cytogenetics of multiple myeloma: a workshop report. *Cancer Research*.

[3] Bergsagel PL, Kuehl WM (2005). Molecular pathogenesis and a consequent classification of multiple myeloma. *Journal of Clinical Oncology*.

[4] Chng WJ, Glebov O, Bergsagel PL, Kuehl WM (2007). Genetic events in the pathogenesis of multiple myeloma. *Best Practice and Research: Clinical Haematology*.

[5] Fonseca R, Blood E, Rue M (2003). Clinical and biologic implications of recurrent genomic aberrations in myeloma. *Blood*.

[6] Fonseca R, Bergsagel PL, Drach J (2009). International myeloma working group molecular classification of multiple myeloma: spotlight review. *Leukemia*.

[7] Zhan F, Huang Y, Colla S (2006). The molecular classification of multiple myeloma. *Blood*.

[8] Shaughnessy JD, Zhan F, Burington BE (2007). Avalidated gene expression model of high-risk multiple myeloma is defined by deregulated expression of genes mapping to chromosome 1. *Blood*.

[9] San-Miguel J, Mateos MV, Gutierrez NC (2009). Risk stratification in the era of novel therapies. *Cancer Journal*.

[10] Smadja NV, Fruchart C, Isnard F (1998). Chromosomal analysis in multiple myeloma: cytogenetic evidence of two different diseases. *Leukemia*.

[11] Debes-Marun CS, Dewald GW, Bryant S (2003). Chromosome abnormalities clustering and its implications for pathogenesis and prognosis in myeloma. *Leukemia*.

[12] Fonseca R, Debes-Marun CS, Picken EB (2003). The recurrent IgH translocations are highly associated with nonhyperdiploid variant multiple myeloma. *Blood*.

[13] Avet-Loiseau H, Attal M, Moreau P (2007). Genetic abnormalities and survival in multiple myeloma: the experience of the Intergroupe Francophone du Myélome. *Blood*.

[14] Kumar SK, Mikhael JR, Buadi FK (2009). Management of newly diagnosed symptomatic multiple myeloma: updated Mayo Stratification of Myeloma and Risk-Adapted Therapy (mSMART) consensus guidelines. *Mayo Clinic Proceedings*.

[15] Carrasco DR, Tonon G, Huang Y (2006). High-resolution genomic profiles define distinct clinico-pathogenetic subgroups of multiple myeloma patients. *Cancer Cell*.

[16] Chesi M, Nardini E, Lim RSC, Smith KD, Michael Kuehl W, Bergsagel PL (1998). The t(4;14) translocation in myeloma dysregulates both FGFR3 and a novel gene, MMSET, resulting in IgH/MMSET hybrid transcripts. *Blood*.

[17] Keats JJ, Reiman T, Maxwell CA (2003). In multiple myeloma, t(4;14)(p16;q32) is an adverse prognostic factor irrespective of FGFR3 expression. *Blood*.

[18] Avet-Loiseau H, Soulier J, Fermand JP (2010). Impact of high-risk cytogenetics and prior therapy on outcomes in patients with advanced relapsed or refractory multiple myeloma treated with lenalidomide plus dexaméthasone. *Leukemia*.

[19] Mateos M, Gutierrez N, Paiva B (2010). Clinical outcome according to both cytogenetic abnormalities (CA) detected by fluorescence in situ hibridization (FISH) and hyperdiploidy assessed by flow cytometry (FCM) in elderly newly diagnosed myeloma patients treated with A Bortezomib-based combination. *Blood*.

[20] San Miguel JF, Schlag R, Khuageva NK (2008). Bortezomib plus melphalan and prednisone for initial treatment of multiple myeloma. *The New England Journal of Medicine*.

[21] Avet-Loiseau H, Malard F, Campion L (2011). Translocation t(14;16) and multiple myeloma: is it really an independent prognostic factor?. *Blood*.

[22] Hoyer JD, Hanson CA, Fonseca R, Greipp PR, Dewald GW, Kurtin PJ (2000). The (11;14)(q13;q32) translocation in multiple myeloma: a morphologic and immunohistochemical study. *American Journal of Clinical Pathology*.

[23] Garand R, Avet-Loiseau H, Accard F, Moreau P, Harousseau JL, Bataille R (2003). t(11;14) and t(4;14) translocations correlated with mature lymphoplasmacytoid and immature morphology, respectively, in multiple myeloma. *Leukemia*.

[24] Fonseca R, Blood EA, Oken MM (2002). Myeloma and the t(11;14)(q13;q32); evidence for a biologically defined unique subset of patients. *Blood*.

[25] Chng WJ, Gonzalez-Paz N, Price-Troska T (2008). Clinical and biological significance of RAS mutations in multiple myeloma. *Leukemia*.

[26] Chng WJ, Kumar S, VanWier S (2007). Molecular dissection of hyperdiploid multiple myeloma by gene expression profiling. *Cancer Research*.

[27] Drach J, Ackermann J, Fritz E (1998). Presence of a p53 gene deletion in patients with multiple myeloma predicts for short survival after conventional-dose chemotherapy. *Blood*.

[28] Zhan F, Colla S, Wu X (2007). CKS1B, overexpressed in aggressive disease, regulates multiple myeloma growth and survival through SKP2- and p27Kip1-dependent and -independent mechanisms. *Blood*.

[29] Tricot G, Barlogie B, Jagannath S (1995). Poor prognosis in multiple myeloma is associated only with partial or complete deletions of chromosome 13 or abnormalities involving 11q and not with other karyotype abnormalities. *Blood*.

[30] Chng WJ, Santana-Dávila R, van Wier SA (2006). Prognostic factors for hyperdiploid-myeloma: effects of chromosome 13 deletions and IgH translocations. *Leukemia*.

[31] Gutiérrez NC, Castellanos MV, Martín ML (2007). Prognostic and biological implications of genetic abnormalities in multiple myeloma undergoing autologous stem cell transplantation: t(4;14) is the most relevant adverse prognostic factor, whereas RB deletion as a unique abnormality is not associated with adverse prognosis. *Leukemia*.

[32] Chiecchio L, Protheroe RKM, Ibrahim AH (2006). Deletion of chromosome 13 detected by conventional cytogenetics is a critical prognostic factor in myeloma. *Leukemia*.

[33] Bergsagel PL, Kuehl WM, Zhan F, Sawyer J, Barlogie B, Shaughnessy J (2005). Cyclin D dysregulation: an early and unifying pathogenic event in multiple myeloma. *Blood*.

[34] Chng WJ, Braggio E, Mulligan G (2008). The centrosome index is a powerful prognostic marker in myeloma and identifies a cohort of patients that might benefit from aurora kinase inhibition. *Blood*.

[35] Fonseca R, San Miguel J (2007). Prognostic factors and staging in multiple myeloma. *Hematology/Oncology Clinics of North America*.

